# The 3,000 rice genomes project

**DOI:** 10.1186/2047-217X-3-7

**Published:** 2014-05-28

**Affiliations:** 1 Institute of Crop Sciences/National Key Facilities for Crop Gene Resources and Genetic Improvement, Chinese Academy of Agricultural Sciences, 12 S. Zhong-Guan-Cun St, Beijing 100081, China; 2 BGI, Bei Shan Industrial Zone, Yantian District, Shenzhen 518083, China; 3 International Rice Research Institute, DAPO 7777, Metro Manila 1301, Philippines

**Keywords:** *Oryza sativa*, Genetic resources, Genome diversity, Sequence variants, Next generation sequencing

## Abstract

**Background:**

Rice, *Oryza sativa* L., is the staple food for half the world’s population. By 2030, the production of rice must increase by at least 25% in order to keep up with global population growth and demand. Accelerated genetic gains in rice improvement are needed to mitigate the effects of climate change and loss of arable land, as well as to ensure a stable global food supply.

**Findings:**

We resequenced a core collection of 3,000 rice accessions from 89 countries. All 3,000 genomes had an average sequencing depth of 14×, with average genome coverages and mapping rates of 94.0% and 92.5%, respectively. From our sequencing efforts, approximately 18.9 million single nucleotide polymorphisms (SNPs) in rice were discovered when aligned to the reference genome of the temperate *japonica* variety, Nipponbare. Phylogenetic analyses based on SNP data confirmed differentiation of the *O. sativa* gene pool into 5 varietal groups – *indica*, aus/boro, basmati/sadri, tropical *japonica* and temperate *japonica*.

**Conclusions:**

Here, we report an international resequencing effort of 3,000 rice genomes. This data serves as a foundation for large-scale discovery of novel alleles for important rice phenotypes using various bioinformatics and/or genetic approaches. It also serves to understand the genomic diversity within *O. sativa* at a higher level of detail. With the release of the sequencing data, the project calls for the global rice community to take advantage of this data as a foundation for establishing a global, public rice genetic/genomic database and information platform for advancing rice breeding technology for future rice improvement.

## Data description

### Purpose of data acquisition

For much of the world’s poor, rice (*O. sativa* L.) is the cereal that provides the majority of daily calories in their staple diet. Rice is also known for its tremendous within-species genetic diversity and varietal group differentiation [1,2]. Rice productivity has more than doubled in recent decades, resulting primarily from the Green Revolution and continued breeding efforts since the 1960s. However, in order to meet the demands imposed by the projected increase in global population, the world’s rice production has to increase by 25% or more by 2030 [3]. This increase has to be achieved under less land, less water and under more severe environmental stresses due to climate change. Thus, accelerated genetic gains are needed in the next few decades to improve yield potential and stability, and grain quality of rice. This requires more complete knowledge of the genetic diversity in the *O. sativa* gene pool, associations of diverse alleles with important rice traits, and systematic exploitation of this rich genetic diversity by integrating knowledge-based tools into rice improvement using innovative breeding strategies [4-6].

To date, a few studies on rice have been undertaken to discover allelic variants through next generation sequencing (NGS) [7-9]. Unfortunately, these studies have been unable to provide a complete picture of the total genetic diversity within the *O. sativa* gene pool, due to either the small sample size of sequenced accessions [7], or the low-coverage sequencing depth of the genomes [8,9]. Here, we report an international effort to extend significantly our understanding of the total genetic diversity within the *O. sativa* gene pool by re-sequencing 3,000 *O. sativa* genomes using IIllumina-based NGS. Our ultimate goal is to establish, through collective efforts by the international scientific community, a public rice database containing genetic and genomic information suitable for advancing rice breeding technology.

### Selection of germplasm

**Figure 1:**
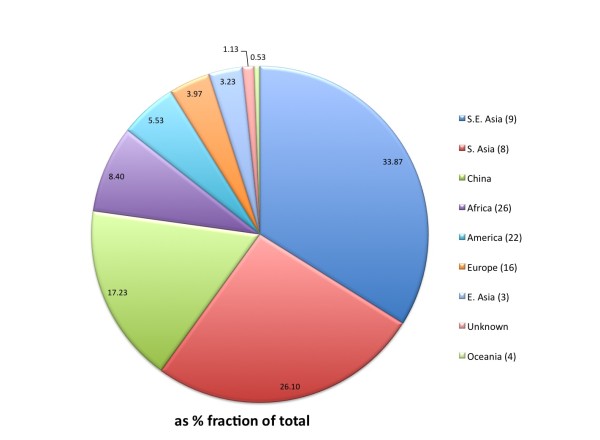
**Geographical distribution of the 3,000 sampled rice accessions from 89 countries (see Additional file**1**: Tables S1A and S1B).** The numbers in the parentheses after each region are the numbers of the countries in the region.

A total of 3,000 germplasm accessions were chosen for sequencing, including 2,466 accessions from the International Rice Genebank Collection (IRGC) at the International Rice Research Institute (IRRI), and 534 accessions from the China National Crop Gene Bank (CNCGB) in the Institute of Crop Sciences, Chinese Academy of Agricultural Sciences (CAAS). The 2,466 accessions (in Additional file 1: Table S1A ) contributed by IRRI represent a panel that was randomly selected from a core collection of 12,000 *O. sativa* accessions that was established by a semi-stratified selection scheme from more than 101,000 rice accessions in the IRGC; taking into account factors, such as the country of origin, eco-cultural type and varietal grouping with even coverage of the name space while limiting potential duplicates from each country, and complemented by specific, nominated entries from IRRI and the Centre de Coopération Internationale en Recherche Agronomique pour le Développement (Cirad). The 534 accessions (in Additional file 1: Table S1B) contributed by CAAS included a mini-core collection of 246 accessions selected from a core collection of 932 accessions established in the same way from the 61,470 *O. sativa* accessions preserved in the CNCGB [10], plus 288 accessions selected based on their isozyme diversity [1], and used as parental lines in the international rice molecular breeding network [2]. Together, the sampled 3,000 rice accessions came from 89 different countries/regions, 77.1% of which are from the centers of rice genetic diversity -Southeast Asia (33.9%), South Asia (25.6%) and China (17.6%) (Figure 1).

Genetic stocks derived from the *O. sativa* accessions were generated for each of the sampled 3,000 rice accessions by one or more cycles of single-seed descent purification under field or screen-house conditions. New accession numbers were assigned to seeds derived from one or more rounds of multiplication starting from a single plant of each source accession. As of March 2013, new accession numbers have been assigned to 1,958 of the IRRI accessions. Purified seeds of the sequenced accessions are (or will be available) from the IRGC or CNCGB as genetic stocks. Information on obtaining seeds from the IRGC can be found at [11] and from the CNCGB at [12].

### Sequencing

Genomic DNA was prepared from bulk harvested leaves of a single young plant for each sampled accession by a modified CTAB method either at IRRI or at CAAS. Genomic DNA samples were then shipped to BGI-Shenzhen and were used to construct Illumina index libraries following the manufacturer’s protocol. Following quality control, at least 3 μg genomic DNA of each sample was randomly fragmented by sonication and size-fractionated by electrophoresis, and DNA fragments of approximately 500 bp were purified. Purified 500 bp DNA fragments from each of the 24 accessions were labeled independently using distinct 6 bp nucleotide multiplex identifiers, followed by pooling prior to library construction for NGS. Each sequencing library was sequenced in six or more lanes on the HiSeq2000 platform and 90 bp paired-end reads were generated. Subsequently, the reads from each sample were extracted based on their unique nucleotide multiplex identifiers as 83 bp reads (90 – 6 – 1, where 1 is the ligation base “T”). To ensure high quality, raw data was filtered by deleting reads having adapter contamination or containing more than 50% low quality bases (quality value ≤ 5).

### Data generation and analyses

#### ***Read alignment and variant identification***

The clean reads were mapped to the temperate *japonica* Nipponbare reference genome – the unified-build release Os-Nipponbare-Reference-IRGSP-1.0 (IRGSP-1.0) [13], using the BWA software with default parameters except for “aln -m 10000 -o 1 -e 10 -t 4”. The alignment results were then merged and indexed as BAM files [14,15]. SNP calling was based on alignment using the Genome Analysis Toolkit 2.0-35 (GATK) and Picard package V1.71 [16]. To minimize the number of mismatched bases for SNP and InDel calling, all reads from each accession were further cleaned by:

(1) deleting the reads that are unmapped to the reference in the alignment result;

(2) deleting duplicate reads;

(3) conducting alignment by the IndelRealigner package in GATK; and

(4) recalibrating realignments using the BaseRecalibrator package in GATK.

SNP and InDel calling for each sample were performed independently using the UnifiedGenotyper package in GATK with a minimum phred-scaled confidence threshold of 50, and a minimum phred-scaled confidence threshold for emitting variants at 10. To ensure the quality of variant calling, the conditions for every site in a genome were set at >20 for mapping quality, >50 for variant quality and >2 for the number of supporting reads for every base.

SNP and InDel calling at the population level (i.e., for all sequenced genomes concurrently) was performed using the UnifiedGenotyper package in the GATK pipeline with 50 for the minimum phred-scaled confidence threshold for variant calling, 30 for the minimum phred-scaled confidence threshold for variant emitting, >20 for the mapping quality, MAF >0.001 for every SNP, and >2 sequence depth for genotypes in every sample. Five independent, randomly selected sets of 200,000 SNPs with minimum missing data were then selected for phylogenetic analysis.

For each of these five sets, distance matrices using the p-distances model were calculated, and Neighbor Joining trees were constructed with 1,000 bootstraps using the TreeBeST software [17]. Consensus trees were exported as Newick format and imported into DarWIN v5.0.158 for topology visualization [18]. For each of the five consensus trees, prior information on variety group designation (based on SSR or isozyme classification) was used to define assignment to one of the five groups – *indica*, aus/boro, basmati/sadri, *japonica* (tropical or temperate). Groupings assigned for each of the five trees were compared using a majority rule criterion (i.e., a minimum of three trees to support the assignment). Those accessions that failed this test were labeled as intermediate types.

### Findings

Using IRGSP-1.0 as the reference, the 3,000 sequenced genomes had an average depth of ~14×, ranging from ~4× to greater than 60×, and yielded a combined total of approximately 17 TB of high quality sequence data. Of the 3,000 entries, 2,322 accessions had >10× sequence depths. When aligned with IRGSP-1.0 using the BWA software, the average genome coverage and mapping rate were 94.0% and 92.5%, respectively. BWA alignment followed by variant calling using GATK identified approximately 18.9 million single nucleotide polymorphisms (SNPs) (Table 1). The distribution of the identified SNPs across different chromosomes varies considerably, with chromosomes 4, 1 and 11 having the highest numbers of SNPs and chromosomes 9, 10 and 5 having the lowest. Most SNPs were detected in intergenic regions and introns, based on comparison with gene annotations provided by MSU v7 [13,19]. Only 18.24% of the detected SNPs occur in exons, of which ~40% are synonymous.

**Table 1:** **Characteristics of the single nucleotide polymorphisms** (**SNPs**) **identified in the 3**,**000 rice genomes when aligned to the reference *japonica *Nipponbare genome IRGSP**-**1.0**

**Chrom.**	**Gene**	**mRNA**	**5’-UTR**	**CDS**	**Intron**	**3’-UTR**	**Intergenic**	**Total**	**Syn**	**Non-syn**	**Total**	**Non-syn/Syn**
Chr1	634,912	630,396	25,880	291,817	286,601	26,098	1,252,989	1,887,901	118,095	173,722	291,817	1.471
Chr2	528,417	524,172	20,087	243,967	238,738	21,380	1,013,475	1,541,892	97,306	146,661	243,967	1.507
Chr3	490,402	487,611	19,899	223,196	224,129	20,387	962,304	1,452,706	88,477	134,719	223,196	1.523
Chr4	730,310	727,473	19,018	388,220	301,071	19,164	1,176,274	1,906,584	160,101	228,115	388,220	1.425
Chr5	489,370	485,848	13,623	257,327	200,307	14,591	867,799	1,357,169	103,723	153,604	257,327	1.481
Chr6	560,506	557,361	16,943	280,933	242,635	16,850	1,023,473	1,583,979	114,625	166,308	280,933	1.451
Chr7	548,266	546,569	16,210	280,994	231,797	17,568	973,670	1,521,936	115,332	165,662	280,994	1.436
Chr8	582,068	580,181	16,396	302,785	244,991	16,009	998,651	1,580,719	124,025	178,759	302,785	1.441
Chr9	436,037	434,440	10,692	222,916	190,025	10,807	763,771	1,199,808	90,299	132,617	222,916	1.469
Chr10	476,710	473,603	11,735	258,013	192,214	11,641	806,940	1,283,650	109,451	148,561	258,013	1.357
Chr11	684,803	681,891	16,642	354,874	291,049	19,326	1,148,735	1,833,538	140,772	214,101	354,874	1.521
Chr12	607,336	603,783	16,549	319,401	251,103	16,730	1,055,044	1,662,380	129,296	190,105	319,401	1.470
ChrUn	19,706	19,706	0	12,615	7,091	0	26,669	46,375	5,819	6,796	12,615	1.168
ChrSy	11,463	11,463	0	7,913	3,550	0	15,043	26,506	3,846	4,067	7,913	1.057
Total	6,800,306	6,764,497	203,674	3,444,971	2,905,301	210,551	12,084,837	18,885,143	1,401,167	2,043,797	3,444,971	1.459

The MSU V7.0 rice gene annotation for 55,986 genes and 66,338 mRNA [13] as a raw gff3 file type was downloaded from the Rice Genome Project Annotation ftp site [19]. Prior to categorization of SNP types, the raw gff3 file was processed 1) to remove all but the primary mRNA transcript and 2) to select the gene models with the highest support in cases where there are overlapping gene models. Hence, SNP characteristics are reported here for 55,107 of the 55,986 gene models. Characteristics of SNPs in pseudogenes or where the reference base is N (unknown or missing) are not reported. Syn = synonymous; Non-syn = non-synonymous.

**Figure 2:**
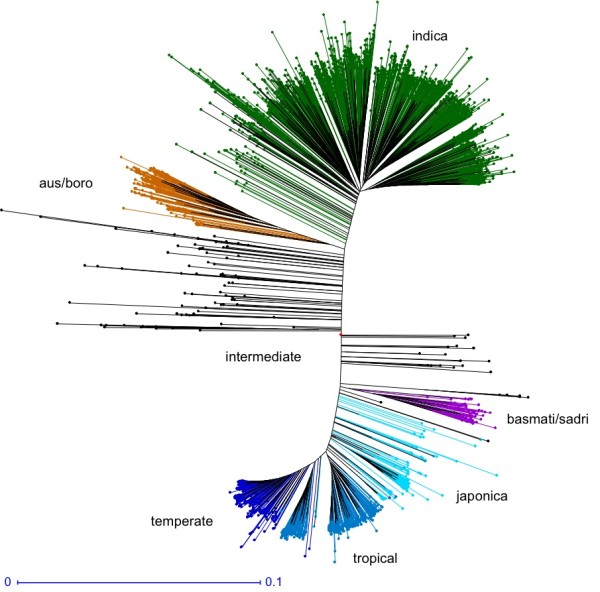
Classification of 3,000 rice accessions into five distinct varietal groups based on 5 sets of 200,000 random sets from the 18.9 million discovered SNP variants.

The phylogenetic analyses revealed clear differentiation of the 3,000 accessions into two major groups – *indica* and *japonica*, two small varietal groups – the aus/boro and basmati/sadri types, plus a small group (134) of intermediate (admixed) types (Figure 2). The *indica* group represented the largest and most diverse group comprising 1,760 (58.2%) accessions in five major subgroups of diverse origins. The *japonica* group contains 843 (27.9%) accessions, which had two well-differentiated subgroups – 388 temperate *japonicas* and 455 tropical *japonicas*. The aus/boro group is composed of 215 accessions and is more closely related to *indica*, while the aromatic basmati/sadri group is more closely related to *japonica* and consists of 68 accessions primarily from South Asia.

## Availability and requirements

### Data availability

The sequencing data of the 3,000 rice genomes project (3K RGP) is now deposited in the *GigaScience* database (*GigaDB*) and has a citable digital object identifier (DOI) [20]. The dataset consists of separate directories for sequences from each of the 3,000 rice genomes. These directories are named by the DNA_UNIQUE_IDs given in Additional file 1: Tables S1A and S1B. If the DNA_UNIQUE_ID contains a space, the space is replaced by an underscore. Each directory contains from 12 to 40 Fastq (fq) files of trimmed, filtered reads that are compressed using GNU zip (gzip, .gz). The dataset consists of about 15.4 terabytes (Tb) of files. Individual data files can be downloaded using tools such as File Transfer Protocol (FTP). In order to obtain the complete dataset, use of FTP is not possible due to the time required for file transfer and bandwidth consumed; other tools will be needed.

**Dataset name:** The 3,000 rice genomes project data

**Operating system:** Platform-independent, UNIX/Linux preferred

**License:** Creative Commons 0 (CC0) public domain dedication (https://creativecommons.org/publicdomain/zero/1.0)

### Data requirements

After download or acquiring, depending on the task, from 8 Gb (reference-guided alignment and variant calling) to 16 Gb (*de novo* genome assembly) or more main memory is needed and from 16 to 64 Gb or more swap space allocated for each pipeline; computation will require from 7 hours (alignment and calling) to 3 days (assembly) per core per pipeline.

## Discussion

This 3,000 rice genomes dataset provides an unprecedented resource for rice genomic research. With access to the genome sequences of the 3,000 accessions representing various varietal types of diverse origins and availability of additional high-quality rice reference genomes, further comparisons can be made among the 3,000 genomes and reference genomes of different rice types. These analyses are expected to uncover the within-species diversity and genome-level population structure of *O. sativa* in great detail. Thus, we hope that this data note will be the beginning of a new round of accelerated discoveries in rice science. Here, we would like to call for an international effort to analyze and mine the dataset. The expected information explosion from follow-up studies of the project will provide a foundation to revolutionize rice genetics and breeding research. Ultimately, this could lead to a more thorough understanding of the molecular, cellular and physiological machineries/networks responsible for the growth and development of rice plants and their responses to various abiotic and biotic stresses.

This data note is accompanied by a 'Commentary’ article, where the intent and plans for the projected uses of the 3,000 rice genomes dataset are further expanded [21]. Through the public release of this dataset, we encourage the global science community to analyze the data and to contribute in building a public rice genetic/genomic database and information platform that will accelerate rice breeding.

## Availability of supporting data

The data set supporting the results of this article is available in the *GigaScience* GigaDB Database [20]. Information on SNP variants will be available on analysis of the population-level genome diversity of the 3,000 rice genomes. Raw sequence data is also available from the SRA at PRJEB6180.

## The 3,000 rice genomes project: participants and affiliations

### Participants by institute

#### *CAAS^1^*

Zhikang Li* Email: zhkli1953@126.com or lizhikang@caas.cn

Bin-Ying Fu Email: fubinying@caas.cn

Yong-Ming Gao Email: gaoyongming@caas.cn

Wen-Sheng Wang Email: wangwensheng02@caas.cn

Jian-Long Xu Email: xujianlong@caas.cn

Fan Zhang Email: zhangfan03@caas.cn

Xiu-Qing Zhao Email: zhaoxiuqing@caas.cn

Tian-Qing Zheng Email: zhentainaqing@caas.cn

Yong-Li Zhou Email: zhouyongli@caas.cn

#### *BGI^2^*

Gengyun Zhang* Email: zhanggengyun@genomics.cn

Shuaishuai Tai Email: taishuaishuai@genomics.org.cn

Jiabao Xu Email: xujiabao@genomics.org.cn

Wushu Hu Email: huwushu@genomics.org.cn

Ming Yang Email: yangming@genomics.org.cn

Yongchao Niu Email: niuyongchao@genomics.org.cn

Miao Wang Email: wangmiao@genomics.org.cn

Yanhong Li Email: liyanhong@genomics.org.cn

Lianle Bian Email: bianlianle@genomics.org.cn

Xuelian Han Email: hanxuelian@genomics.org.cn

Jun Li Email: lijun3@genomics.org.cn

Xin Liu Email: liuxin@genomics.org.cn

Bo Wang Email: wangbo@genomics.org.cn

#### *IRRI^3^*

Kenneth L. McNally* Email: k.mcnally@irri.org

Ma. Elizabeth B. Naredo Email: e.naredo@irri.org

Sheila Mae Q. Mercado Email: s.mercado@irri.org

Myla Christy Rellosa Email: m.rellosa@irri.org

Renato A. Reaño Email: r.reano@irri.org

Grace Lee S. Capilit Email: g.capilit@irri.org

Flora C. de Guzman Email: f.deguzman@irri.org

Jauhar Ali Email: j.ali@irri.org

N. Ruaraidh Sackville Hamilton Email: r.hamilton@irri.org

Ramil P. Mauleon Email: r.mauleon@irri.org

Nickolai N. Alexandrov Email: n.alexandrov@irri.org

Hei Leung Email: h.leung@irri.org

## Abbreviations

3K RGP: 3,000 rice genomes project; BGI: Beijing Genomics Institute Shenzhen; CAAS: Chinese Academy of Agricultural Sciences; Cirad: Centre de Coopération Internationale en Recherche Agronomique pour le Développement; CNCGB: China National Crop Gene Bank; GATK: Genome Analysis Toolkit; IRGC: International Rice Genebank Collection; IRRI: International Rice Research Institute; NGS: Next generation sequencing.

## Competing interests

The authors declare that they have no competing interests.

## Authors’ contributions

ZKL, GZ, KLM, and HL designed the project; KLM and NRSH selected the IRRI materials; KLM, MEBN, SMQM, RAR, MCR, GLSC, and FCG prepared and curated the IRRI materials; ZKL, WSW, YMG, TQZ, JLX, XQZ, FZ, YLZ and JA selected and prepared the CAAS materials; MEBN, SMQM and MCR prepared the IRRI DNAs; WSW, YLZ, BYF, TQZ prepared the CAAS DNAs; WH, MY, YN, MW, YH, LB, XL, BW, JL, JX, ST and XL performed the sequencing and NGS data analyses; SS and KLM performed the phylogenetic analyses; ZKL, GZ, KLM, NNA, RPM, HL and JA wrote the manuscript. All authors have read and approved the final manuscript.

## Supplementary Material

Additional file 1: Table S1A Information for the 2,466 rice accessions from the International Rice Genebank Collection at the International Rice Research Institute. **Table S1B.** Information for the 534 rice accessions from the China National Crop Genebank and the CAAS working collections. Click here for file
